# Role of homovanillic acid esters in the regulation of skin inflammatory pathways and their effect on tight junction protein expression

**DOI:** 10.3389/fphar.2025.1629941

**Published:** 2025-07-21

**Authors:** Maria Fernanda Cervantes Recalde, Elena Zoe Bogensperger, Joachim Hans, Dominik Stuhlmann, Veronika Somoza, Barbara Lieder

**Affiliations:** ^1^ Institue of Physiological Chemistry, Faculty of Chemistry, University of Vienna, Vienna, Austria; ^2^Vienna Doctoral School in Chemistry (DoSChem), University of Vienna, Vienna, Austria; ^3^ Symrise AG, Holzminden, Germany; ^4^Leibniz Institute of Food Systems Biology, Technical University of Munich, Freising, Germany; ^5^Institute of Clinical Nutrition, University of Hohenheim, Stuttgart, Germany

**Keywords:** skin, inflammation, TRPV1, claudin 1, homovanillic acid ester

## Abstract

**Introduction:**

In the context of epidermal inflammation, the inflammatory response not only involves the release of inflammatory cytokines like interleukin 8 (IL-8), but also modulation of tight junction protein expression levels. Previous studies showed that the tight junction protein claudin 1 (CLDN1) is upregulated during tumor necrosis factor α (TNFα)-induced inflammation by capsaicin in keratinocytes in a transient receptor potential channel vanilloid 1 (TRPV1)-dependent manner. However, the caveat with TRPV1 ligands is the undesired pain response elicited by the activation of neuronal TRPV1 channels. In this study, we hypothesized that also less or non-pungent homovanillic acid esters as structural analogs of capsaicin target CLDN1 upregulation during inflammation.

**Methods:**

We aimed to identify beneficial structural characteristics by selecting homovanillic acid esters with different aliphatic tail structures and screening them for CLDN1 upregulation at early stages of TNFα-induced inflammation in basal keratinocytes.

**Results:**

CLDN1 expression was upregulated independently of TRPV1 by compounds with a tail of 5 or 6 C-atoms, regardless of the presence of ramifications and double bonds with a maximum fold change of 2.05 ± 0.22 against control. The induction of CLDN1 expression was accompanied by increased expression of the differentiation marker involucrin (IVL).

**Discussion:**

The results suggest that the homovanillic ester-induced CLDN1 upregulation is a result of increased differentiation of the basal keratinocytes towards the keratinocyte morphology present in the stratum granulosum (SG), where tight junctions are formed. In conclusion, homovanillic acid esters with a 5 or 6 C-atom long aliphatic chain induced CLDN1 expression, thereby stimulating keratinocyte differentiation, independent from TRPV1 activation.

## 1 Introduction

As the outermost layer of the skin, the epidermis serves as the primary point of contact for the body with bacterial infections, allergens, and other harmful external stimuli. When cells in the epidermis encounter these threats, the activation of receptors trigger signaling pathways that release a variety of inflammatory mediators rapidly (Chen et al., 2018; Banno et al., 2004). These mediators attract immune cells to the site of infection or injury, where they become activated to clear away dead cells and eliminate pathogens. TNFα is one of the key factors driving these inflammatory processes by orchestrating pathways that stimulate the release of inflammatory cytokines, regulate apoptosis, and induce tissue remodeling (Banno et al., 2004). Ultimately, the successful resolution of epidermal inflammation requires the restoration of keratinocyte barrier function (Chen et al., 2018). Claudin 1 (CLDN1) plays a critical role in skin barrier function by forming the intercellular strands of the tight junctions (TJ) that regulate permeability in the *stratum granulosum* (SG) as well as maintain *stratum corneum* (SC) integrity (Kirschner et al., 2013; Sugawara et al., 2013; Lynn et al., 2020). A differentiation-driven gene expression program in basal epidermal layer keratinocytes ensures CLDN1 presence in the SG, in conjunction with increasing expression of the differentiation marker involucrin (*IVL*) and halting the production of basal layer markers like keratin 14 (*KRT14*) (Watt, 1983; Fuchs, 1993). Tumor necrosis factor α (TNFα) favors this differentiation program via the nuclear factor kappa beta (NF-κB) pathway (Banno et al., 2004).

In a previous study, the effect of an increase in TNFα levels on the expression of *CLDN1* was characterized using an inflammatory model of basal keratinocyte morphology. Due to their ability to differentiate in culture, the HaCaT keratinocyte cell line provided an adequate model for examining the impact of inflammation on differentiation-dependent cellular structures such as tight junctions (TJs) (Wilson, 2014). Specifically, incubation of HaCaT cells with 20 ng/mL TNFα for 48 h dose-dependently promoted *CLDN1* expression, demonstrating that the inflammatory response impacts the production of TJ proteins already in lower epidermal layers to prevent a loss of barrier function (Cervantes Recalde et al., 2024). Additionally, TNFα-induced *CLDN1* expression was enhanced by capsaicin and inhibited by capsazepine, indicating that TRPV1 activation also participates in the regulation of TJ proteins during inflammation (Cervantes Recalde et al., 2024). However, although capsaicin’s bioactivity is often attributed to its interaction with the TRPV1 channel, it is important to consider that capsaicin and structurally analog compounds of capsaicin may also trigger responses through TRPV1-independent mechanisms. Examples of this include the capsaicin-mediated inhibition of natural killer cell cytotoxicity, anti-tumor activity of capsaicin in oral cancer and counteraction of lipopolysaccharide (LPS)-induced hyperthermia in chicken (Gonzales et al., 2014; Nikami et al., 2008; Kim et al., 2014). This raises the question whether the promotion of *CLDN1* during TNFα-induced inflammation can also be elicited by less or non-pungent structural analogs of capsaicin and if these effects are TRPV1 dependent.

From a structural perspective, the affinity for the TRPV1 channel can be fine-tuned by introducing modifications in three key pharmacophores that define structure-activity relationships in TRPV1 ligands: the aromatic “head” binding through hydrogen bonds the vanilloid pocket, a polar “neck”, and the aliphatic “tail” providing further biding affinity through Van der Waals interactions with surrounding residues (Caballero, 2022). Modifications to these regions will likely alter the molecule’s biological activity (Yang et al., 2015; Elokely et al., 2016). Capsaicin analogs with structural modifications have been demonstrated to have a reduced ability to activate the TRPV1 receptor in skin, eye and the oral cavity of mice (Meotti et al., 2014). For example, the direct replacement of the amide bond for an ester bond in the non-pungent analog capsiate changes the EC_50_ for TRPV1 activation from 0.099 µM (capsaicin) to 0.290 µM and evokes only a fraction of capsaicin’s pungency (Meotti et al., 2014; Kobata et al., 1998). This modification also diminished other TRPV1-induced effects such as intestinal fatty acid uptake or thermoregulation of gene expression in white adipose tissue (Lieder et al., 2019; Baskaran et al., 2018). The reduced affinity for the TRPV1 channel is not restricted to modifications in the neck pharmacophore of the ligand, as differences in the aliphatic tail also have consequences for the binding affinity (Chen et al., 2019).

**FIGURE 1:**
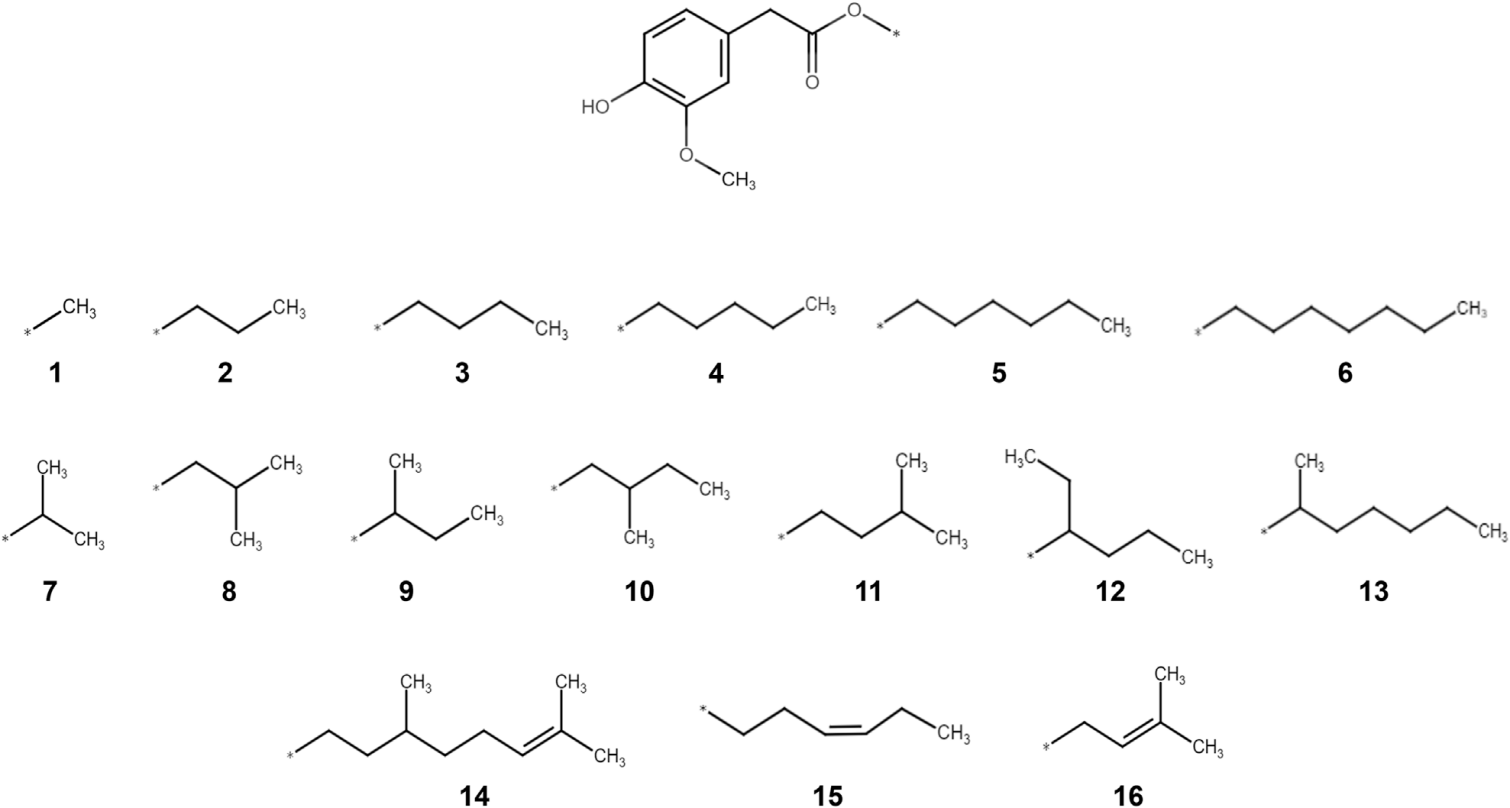
Molecular structures of the 16 homovanillic acid esters (**1**-**16**) used in the present study. The different homovanillic acid esters share the homovanillic ring “head” structure and an ester bond in the “neck” of the molecule. The different aliphatic tails are separately drawn, grouped according to chain length (**1**-**6**), ramifications (**7**-**13**) and double bonds (**14**-**16**).

Homovanillic acid esters have been characterized in the past as capsaicin or resiniferatoxin (RTX) analogues with reduced pungency to clarify the involvement of TRPV1 in pain and cell apoptosis (Macho et al., 2000; Liu et al., 1998; Appendino et al., 1996). These molecules have ester bonds instead of the amide bond characteristic in capsaicin and their tail pharmacophore can be synthetically modified with different moieties according to the needs of the researcher (Appendino et al., 1996). We, therefore, studied here the potential of less or non-pungent homovanillic acid esters as structural analogs of capsaicin to target CLDN1 upregulation in keratinocytes during inflammation. We aimed to achieve a better understanding of the structural characteristics that regulate *CLDN1* expression in keratinocytes by evaluating the use of homovanillic acid esters with varying structural modifications in the tail pharmacophore characteristic of TRPV1 ligands (Figure 1). We hypothesized that homovanillic acid esters with defined structural features in their aliphatic tail induce *CLDN1* expression in basal layer keratinocytes independent of TRPV1. Changes in inflammation and differentiation markers (i.e., chemokine (CXC motif) ligand 8 (*CXCL8*), *IVL* and *KRT14*) were documented along with *CLDN1* expression in a basal layer keratinocyte inflammation model and structure-activity relationships (SAR) were drawn to identify *CLDN1* promoting structures.

## 2 Materials and methods

### 2.1 Chemicals

All chemicals used in this study were obtained at a ≥95% purity. TNFα (abcam) and capsazepine (Sigma Aldrich) were commercially purchased. Compounds **1** to **16** (Figure 1) were synthesized as described previously (Lieder et al., 2019). Treatment of the cell model with compounds **1**-**16** was done at a concentration of 10 µM using 0.1% dimethyl sulfoxide (DMSO) as solvent (Cervantes Recalde et al., 2024). Capsazepine was dissolved in 0.1% DMSO at a concentration of 1 µM and used in combination with the respective treatments with a resulting concentration of 0.2% DMSO. TNFα was used at a concentration of 20 ng/mL using double distilled water (ddH_2_O) as a solvent.

### 2.2 Cell culture

HaCaT keratinocyte cells (Cell Lines Service) were cultured under low calcium conditions (Wilson, 2014) using the keratinocyte growth medium 2 kit (PromoCell) that included basal medium supplemented with 5 μg/mL insulin, 0.33 μg/mL hydrocortisone, 0.004 mL/mL bovine pituitary extract, 10 µ/mL transferrin, 0.06 mM CaCl_2_, 0.125 ng/mL epidermal growth factor and 0.39 μg/mL epinephrine. Furthermore, a penicillin/streptomycin mix (Sigma Aldrich) was added at a concentration of 1% (v/v). Cells were cultured in a humidified and sterile incubator at 37°C at 5% CO_2_.

### 2.3 Treatment of cells with homovanillic acid esters and TNFα induction

The screening of the 16 homovanillic acid esters was performed on HaCaT keratinocytes seeded at a density of $1.5\times 10^{4}$ cells/cm^2^ and incubated under sterile conditions for 4 days before treatment with 10 µM of compounds **1**-**16** for 24 h (Cervantes Recalde et al., 2024). This was followed by a 6 h treatment using 20 ng/mL TNFα. Compounds **4**, **14** and **15** were then selected to be tested in combination with 1 µM capsazepine (TRPV1 antagonist) for 24 h followed by 6 h incubation with 20 ng/mL TNFα.

### 2.4 Cell viability assay

The cell viability of the HaCaT keratinocytes was verified after use of compounds **1**-**16** at a concentration of 10 µM. This was done using an 3-(4,5-dimethylthiazol-2-yl)-2,5-diphenyl tetrazolium bromide (MTT) assay (Lieder et al., 2020). After a 24 h treatment with the different compounds (see 2.3) the medium was replaced with a 1 mg/mL MTT solution (Roth) in medium and incubated at 37°C and 5% CO_2_ for 15 min. The MTT solution was replaced with 100% DMSO for the dilution of the newly formed formazan crystals. The absorbance was measured simultaneously at 570 and 650 nm (reference wavelength) with a Spark^®^ Multimode Microplate Reader (Tecan, Switzerland). Cell viability was calculated as percentage of the solvent control.

### 2.5 RNA isolation and RT-PCR

Sample RNA was isolated using the Monarch Total RNA Miniprep Kit (New England Biotechnologies) as stated in the manufacturer’s protocol. The quantification of the RNA concentration was carried out using a NanoQuant plate and a Spark^®^ Multimode Microplate Reader at an absorbance of 260 nm combined with the assessment of the purity and integrity of the RNA using the 260/230 and 260/280 absorbance ratios. RNA reverse transcription was performed using 0.5 µg of the obtained RNA and processing it with the LunaScript RT SuperMix Kit (New England Biotechnologies) following the instructions provided by the manufacturer. The transcription took place in a thermal cycler C1000 Touch™ (BioRad) and the resulting cDNA was diluted 1:5 using RNase free water to be used as a template for real time qPCR (RT-qPCR) amplification. The cDNA templates were mixed with Luna Universal qPCR Master Mix (New England Biotechnologies) and the respective primer pairs (Sigma Aldrich) for *CLDN1*, *CXCL8, IVL*, *KRT14*, glyceraldehyde-3-phosphate dehydrogenase (*GAPDH*) and hypoxanthine phosphoribosyltransferase 1 (*HPRT1*) (Table 1). RT-qPCR measurement was done in a fluorescent quantitative detection system FQD-96A (Bioer) and the data analysis performed in LinRegPCR (version 2020.0) (Untergasser et al., 2021). The mRNA sample concentration (N_0_ values) was calculated using the C_T_ values and the PCR efficiency followed by normalization against the geometric mean of the reference genes (*GADPH* and *HPRT1*).

**TABLE 1:** Primer pairs used for the characterization of the gene expression induced by TNFα treatment.

Gene	Primer (forward)	Primer (reverse)	References
*CLDN1*	CCA​GTC​AAT​GCC​AGG​TAC​GAA	CAC​ACG​TAG​TCT​TTC​CCG​CT	Cervantes Recalde et al. (2024)
*CXCL8*	ACT​GAG​AGT​GAT​TGA​GAG​TGG​AC	AAC​CCT​CTG​CAC​CCA​GTT​TTC	Soares et al. (2014)
*IVL*	TCC​TCC​AGT​CAA​TAC​CCA​TCA​G	CAG​CAG​TCA​TGT​GCT​TTT​CCT	Yoshioka et al. (2017)
*KRT14*	TGA​GCC​GCA​TTC​TGA​ACG​AG	GAT​GAC​TGC​GAT​CCA​GAG​GA	Gao et al. (2018)
*GADPH*	AGG​TCG​GAG​TCA​ACG​GAT​TTG	GGG​GTC​ATT​GAT​GGC​AAC​AAT​A	Walker et al. (2017)
*HPRT1*	CCT​GGC​GTC​GTG​ATT​AGT​GA	CGA​GCA​AGA​CGT​TCA​GTC​CT	Lieder et al. (2020)

### 2.6 *In vitro* IL-8 ELISA assay

IL-8 cytokine release was assessed using a Human IL-8 ELISA Kit (abcam). HaCaT cells at a density of $3\times 10^{4}$ cells/cm^2^ and treated as specified in Section 2.3. After treatment completion, the medium was removed from the wells, centrifuged at 2,000 *g* for 10 min and diluted 1:10 with the Sample Diluent NS included in the IL-8 ELISA kit. Thereafter the samples were added to the SimpleStep Pre-Coated 96-Well Microplate in the kit and it was proceeded as described by the manufacturer. The optical density (OD) was recorded with a Spark^®^ Multimode Microplate Reader at 450 nm.

### 2.7 Computational determination of physicochemical descriptors

Analysis on the structural characteristics of compounds **1** to **16** was done using the KNIME analytics platform 5 and the RDKit node was used to extract physicochemical descriptor information on the structures of compounds **1** to **16** (Berthold et al., 2008; Landrum et al., 2025). The descriptors were molecular weight in g/mol, SlogP, Labute’s approximate surface area (ASA), standard molecular refractivity (SMR), number of rotatable bonds, number of atoms and bond count. They were used for SAR analysis in combination with the experimentally obtained data of *CLDN1* relative gene expression. Microsoft Excel was additionally used for tabulation and analysis of the data.

### 2.8 Statistical analysis

Statistical analysis was performed using Microsoft Excel and GraphPad Prism Version 10.1.1 software. Data is presented as mean +standard error of mean (SEM). Outliers were excluded after performing a ROUT test with a cut-off value (Q) of 5% (Motulsky and Brown, 2006). Gaussian distribution of the data was tested using a Shapiro-Wilk normality test. An F-test was used to check for equal variances between two groups and a Brown-Forsythe analysis of variance (ANOVA) test was applied when a larger group set was evaluated. Significant differences between two groups were evaluated using a Welch’s t-test or a Mann-Whitney t-test for non-parametric comparisons. Statistical analysis in comparisons between larger sets was achieved using a Brown-Forsythe and Welch ANOVA together with the Dunnett’s T3 multiple comparisons *post hoc* test. P-values of less than 0.05 were considered significant. Correlations were performed using a Spearman correlation between the average technical replicates of the 5 biological replicates for each treatment.

## 3 Results

### 3.1 The aliphatic tail in homovanillic acid esters can be modified to promote *CLDN1* and *CXCL8* gene expression

For the evaluation of structural characteristics important for the *CLDN1* upregulating effect, a set of 16 compounds was selected based on the “head, neck and tail” model previously specified (Caballero, 2022). All compounds shared the same homovanillic ring “head” and an ester bond in the “neck” of the molecule, but the aliphatic tails of the different compounds varied according to chain length (**1**-**6**), ramification points (**7**-**13**) and double bonds (**14**-**16**) (Figure 1). Based on previous studies (Cervantes Recalde et al., 2024), the 16 compounds were applied for 24 h at a 10 µM concentration followed by a TNFα treatment for 6 h, respectively. Detrimental effects of the treatments on cell viability were excluded using MTT assays. None of the compounds showed adverse effects on the viability of HaCaT keratinocytes (Supplementary Figure S1) and baseline levels of *CLDN1* and *CXCL8* expression after TNFα induction were established (Supplementary Figure S2).

**FIGURE 2:**
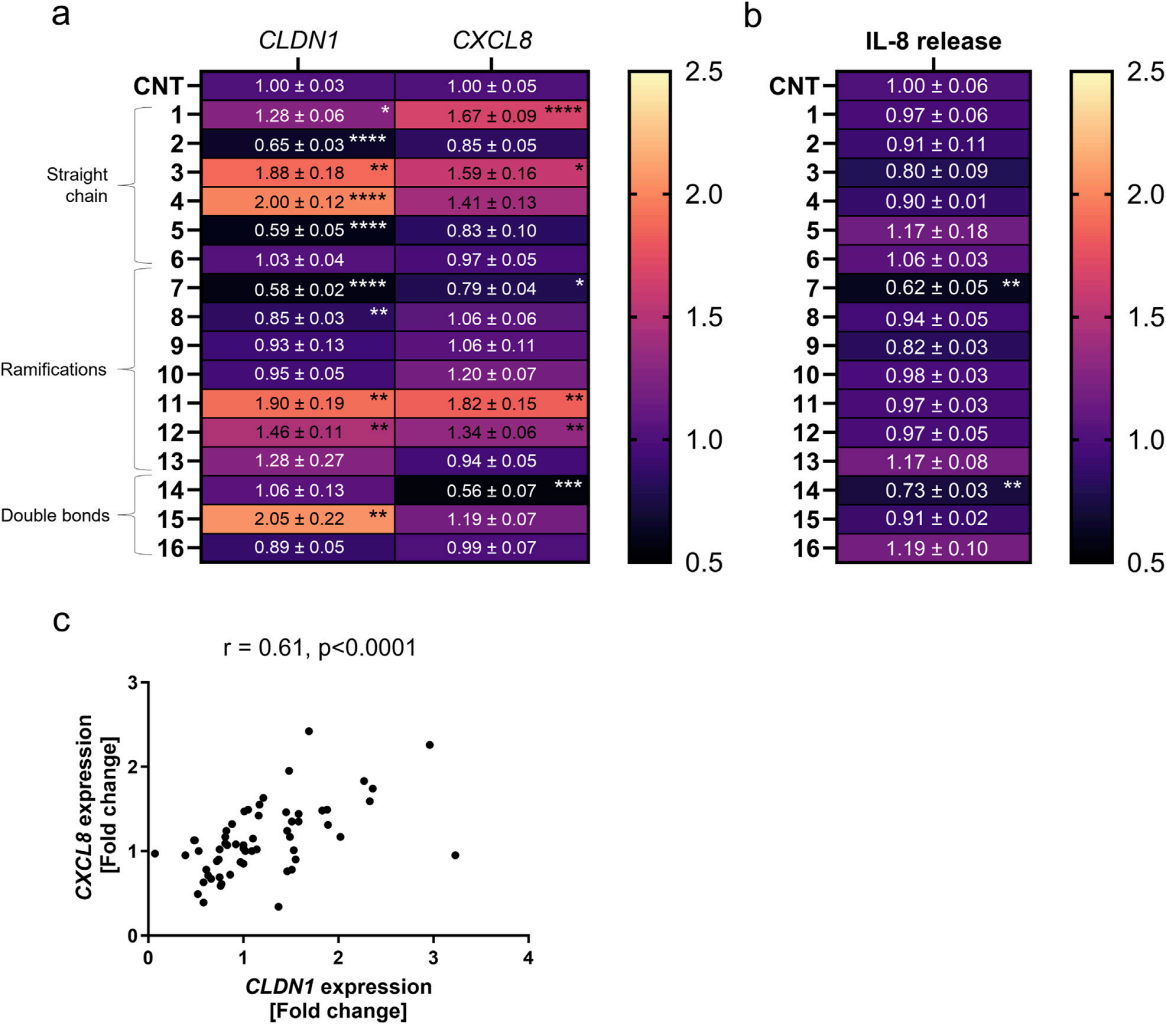
*CXCL8* and *CLDN1* gene expression is differentially influenced by pre-treatment with various homovanillic esters dependent on the structure of the side chain. HaCaT cells were pre-treated with 10 µM of compounds **1**–**16** before TNFα treatment for 6 h. **(a)** *CXCL8* and *CLDN1* gene expression in cells pre-treated with compounds **1**-**6** of increasing side chain length, compounds **7**-**13** with side chain ramifications and compounds **14**-**16** with side chains with double bonds. **(b)** IL-8 release in cells pre-treated with compounds **1**-**16**. **(c)** Moderate correlation (r = 0.61) between the expression of both *CXCL8* and *CLDN1*. *CXCL8* and *CLDN1* gene expression were measured using RT-qPCR and IL-8 release was measured using ELISA. Data presented as the fold change to the non-pretreated TNFα control (CNT) in a magma heatmap with an upper fold change limit of 2.5 (light orange) and a lower fold change limit of 0.5 (black). [Statistics: mean + SEM; technical replicates: 3, biological replicates: 4; Brown-Forsythe and Welch’s ANOVA with Dunnett’s T3 multiple comparisons *post hoc* test, *p < 0.05; **p < 0.01, ***p < 0.001, ****p < 0.0001; **(c)** Spearman correlation].

First, *CXCL8* gene expression was measured and the related IL-8 cytokine release. Four compounds increased *CXCL8* expression compared to cells only treated with TNFα for 6 h. Compounds **1**, **3**, **11** and **12** caused a relative fold increase of 1.67 ± 0.09, 1.59 ± 0.16, 1.83 ± 0.15 and 1.34 ± 0.06 as compared to the TNFα control set to one (Figure 2a). Compounds **7** and **14** were the only compounds which downregulated *CXCL8* gene expression by a fold change of 0.79 ± 0.04 and 0.56 ± 0.07 against control, respectively (Figure 2a). When testing the IL-8 cytokine release, **7** and **14** reduced the release caused after TNFα treatment to 0.62 ± 0.05 and 0.73 ± 0.03-fold (Figure 2b). The expression of *CLDN1* was significantly downregulated by **2** and **5** in the straight chain group by a fold change of 0.65 ± 0.03 and 0.59 ± 0.05 against control as well as **7** and **8** in the ramifications group by a fold change of 0.58 ± 0.16 and 0.85 ± 0.03, respectively (Figure 2a). However, compounds in all three groups, **1**, **3**, **4**, **11**, **12** and **15,** upregulated the expression of *CLDN1* in comparison to the TNFα control by a fold change of 1.28 ± 0.06, 1.88 ± 0.18, 2.00 ± 0.12, 1.90 ± 0.19, 1.46 ± 0.11 and 2.05 ± 0.22, respectively (Figures 2a). Therefore, structure differences in the tail of the tested homovanillic acid esters were shown to modulate *CXCL8* and *CLDN1* expression. The *CXCL8* and *CLDN1* gene expression correlated with a r_s_ value of 0.61 (p < 0.0001) (Figure 2c). Consequently, differences in the aliphatic tail of the selected homovanillic acid esters resulted in similar changes in *CLDN1* and *CXCL8* gene expression during TNFα-induced inflammation when used as a 24 h pre-treatment.

### 3.2 Structural characteristics in the homovanillic ester aliphatic tail can be modified to promote *CLDN1* gene expression and reduce *CXCL8* expression or IL-8 release

We then investigated how structural modifications of the homovanillic acid ester aliphatic tail—specifically chain length, branching, and double bonds—affect *CXCL8* and *CLDN1* gene expression (Figure 2a). The number of C-atoms in the aliphatic tail is provided in Table 2. The longest chains had 10 C-atoms in total, with the longest main chain comprising 8 C-atoms. Any tendencies in the structural features were evaluated considering all compounds and within individual groups classified by the chain length (**1**-**6**), ramification points (**7**-**13**) and double bonds (**14**-**16**).

**TABLE 2:** Physicochemical descriptors of compounds 1-16 obtained through the RD kit in the KNIME analytics platform.

Group	Compound	C-atoms in main chain	Total C-Atoms in chain	Molecular weight [g/mol]	SlogP	SMR	Labute ASA	Number of rotatable bonds	Number of atoms	Bond count	Number of stereo-centers
Straight chain	**1**	1	1	196.20	1.12	50.38	82.07	3	26	14	0
	**2**	3	3	224.26	1.90	59.61	94.80	5	32	16	0
	**3**	4	4	238.28	2.29	64.23	101.17	6	35	17	0
	**4**	5	5	252.31	2.68	68.85	107.53	7	38	18	0
	**5**	6	6	266.34	3.07	73.46	113.90	8	41	19	0
	**6**	7	7	280.36	3.46	78.08	120.26	9	44	20	0
Ramifications	**7**	2	3	224.26	1.89	59.59	94.80	4	32	16	0
	**8**	3	4	238.28	2.14	64.16	101.17	5	35	17	0
	**9**	3	4	238.28	2.29	64.21	101.17	5	35	17	1
	**10**	4	5	252.31	2.53	68.78	107.53	6	38	18	1
	**11**	4	5	252.31	2.53	68.78	107.53	6	38	18	0
	**12**	4	6	266.34	3.07	73.44	113.90	7	41	19	1
	**13**	6	7	280.36	3.46	78.06	120.26	8	44	20	1
Double bonds	**14**	8	10	320.43	4.26	91.77	138.67	9	51	23	1
	**15**	6	6	264.32	2.84	73.37	113.21	7	39	19	0
	**16**	4	5	250.29	2.45	68.75	106.84	5	36	18	0

^a^ Compounds that only increased *CLDN1* expression significantly compared to the TNFα, only control are highlighted in green and compounds that increased both *CXCL8* and *CLDN1* expression significantly are highlighted in red.

Treatment with compounds **3**, **11** and **12** upregulated *CXCL8* expression by a fold change of 1.59 ± 0.16, 1.82 ± 0.15 and 1.34 ± 0.06 compared to the TNFα control. These three compounds share the common feature of having 4 C-atoms in the main chain of the homovanillic acid ester tail. Compounds **10** and **4** also had the tendency to increase *CXCL8* expression, albeit not significant, and shared in common with compound **11** the number of five total C-atoms in the tail structure. *CXCL8* expression was upregulated by compounds with or without ramifications in the aliphatic chain but double bonds were not present in any of the compounds that increased *CXCL8* expression. On the other hand, downregulation of *CXCL8* was achieved when the cells were treated with compounds **7** (0.79 ± 0.04) and **14** (0.56 ± 0.07). These compounds did not share features other than the presence of ramifications in the chain. Both compounds also reduced IL-8 release by fold changes of 0.62 ± 0.05 and 0.73 ± 0.03 against control. Notably, only compounds that downregulated *CXCL8* expression had a consequential effect on IL-8 release. The lowest value in the IL-8 release was obtained with the use of compound 14 which had the longest tail structure with 8 C-atoms in the main chain, 10 C-atoms in total. Therefore, *CXCL8* expression was increased by compounds with predominantly 4–5 C-atoms in the aliphatic tail and downregulation of this gene did not show any distinctive pattern for structural features.

*CLDN1* expression increased with the total number of C-atoms until the homovanillic acid ester tail had 5 C-atoms (straight chain group) or 6 C-atoms (ramifications or double bond groups) reaching the highest fold change versus TNFα control in compound **15** (2.05 ± 0.22) with 6 C-atoms and double bond in the aliphatic chain (Figure 2a). The lowest values were seen for aliphatic tails with 3 (C3) C-atoms. In the straight chain group, *CLDN1* expression reached its highest values after treatment with **4** (C5) with a fold change of 2.00 ± 0.12 against the TNFα control. Within the ramification group, compound **11** (C5) induced the highest *CLDN1* expression with a 1.90 ± 0.20 -fold increase in comparison to non-pre-treated cells followed by **12** (C6) and **13** (C7) with 1.46 ± 0.11 and 1.28 ± 0.27, respectively. Finally, the highest *CLDN1* expression within the double bond group was induced by compound **15**, with 6 C-atoms in the chain and one double bond, as it reached a 2.05 ± 0.22 -fold increase in comparison to the TNFα control. Thus, among the homovanillic esters tested in this study, the highest *CLDN1* expression was induced with compounds with 5 or 6 C-atoms in the aliphatic tail.

Modulation of the *CLDN1* gene expression did not follow a reproducible pattern when considering differences in tail structure for homovanillic acid esters from the ramifications (**7**-**13**) and the double bond (**14**-**16**) groups. Within the ramification group, the lowest values in *CLDN1* expression are found after treatment with compounds **7** (0.58 ± 0.02) and **8** (0.85 ± 0.03), which have smaller side chains, and the largest after treatment with compounds **12** (1.46 ± 0.11) and **11** (1.90 ± 0.20), with 6 and 5 total C-atoms in the side chain. For compounds **8** and **10**, the ramification was positioned in the second C-atom after the ester bond and both had similar fold change difference to the TNFα for *CLDN1* expression with 0.85 ± 0.03 and 0.95 ± 0.05, respectively. For the isomers **10** and **11,** the ramification changes from the second C-atom (**10**) after the ester bond to the third (**11**), but the fold change in *CLDN1* expression is twice as high after treatment with **11**. When considering the double bond group (**14**-**16**), the highest *CLDN1* expression change was caused by **15** (2.05 ± 0.22) with 6 C-atoms in the chain and a double bond in third position after the ester bond. Compound **16** (0.89 ± 0.05), which has the same number of C-atoms as compound **11** (1.90 ± 0.20) but has a double bond in the second position after the ester bond, did not increase *CLDN1* expression. Thus, ramifications or double bonds are no determinant for increased *CLDN1* expression, although addition of ramifications or double bonds in the third position after the ester bond are characteristic of the homovanillic acid esters in the collection that favor upregulation of *CLDN1* compared to the TNFα control.

Lastly, several physicochemical factors were calculated using the RDKit node on the KNIME analytics platform 5 (Table 2). Because the compound that downregulated CXCL8 expression the most was the largest compound, **14**, it follows that the molecular weight, SlogP, SMR, Labout ASA, number of rotatable bonds, number of atoms, bond count and largest chain size would be more likely to favor downregulation of the gene. Compounds **6**, **13** and **14** were the largest compounds within each category (unbranched, ramified and double bonded) but only compound **14** downregulated *CXCL8* significantly. Nevertheless, no compound with a molecular weight higher than 280.36 g/mol upregulated *CXCL8* expression. Among the factors that did not show any distinct patterns related to changes in *CLDN1* expression were the molecular weight, the SlogP, the SMR, the Labout ASA, the number of atoms, the bond count and the number of stereocenters (Table 2). However, within the category of the number of rotatable bonds compounds with 6 and 7 rotatable bonds, except for **10,** produced the highest fold change increase in *CLDN1* expression compared to the control (Table 2). Thus, the number of rotatable bonds is an important characteristic of the homovanillic acid esters in this study that contributed to the upregulation of *CLDN1* gene expression.

### 3.3 The TRPV1-inhibitor capsazepine did not affect the effects elicited by homovanillic acid esters

**FIGURE 3:**
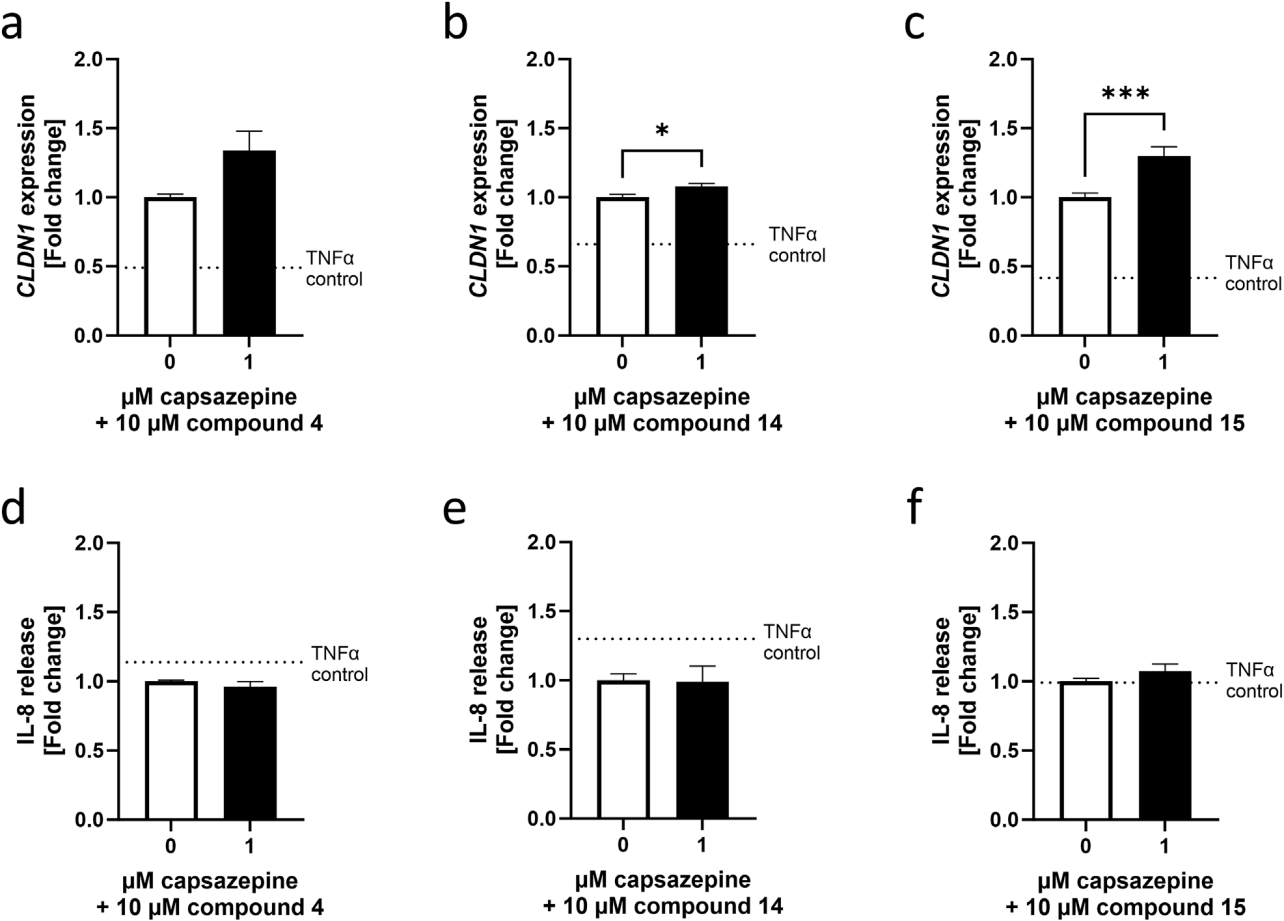
Homovanillic ester effect on *CLDN1* expression is independent from TRPV1 channel. A 10 µM pre-treatment of HaCaT cells with compound **4**, compound **14** or compound **15** in combination with 1 µM TRPV1 antagonist capsazepine was performed in HaCaT cells before induction of inflammation with 20 ng/mL TNFα. Combination of capsazepine with compound **4** resulted in no significant changes in **(a)** *CLDN1* gene expression or **(d)** IL-8 release compared to cells pre-treated without capsazepine. Combination of capsazepine with compound **14** resulted in **(b)** significantly increased *CLDN1* expression but **(e)** no alteration of IL-8 release in comparison to the samples that did not receive capsazepine in the pre-treatment. Finally, *CLDN1* was significantly increased in samples pre-treated with **(c)** a combination of compound **15** and capsazepine compared to samples only treated with compound **15** but there was no significant change in IL-8 release **(f)**. White bars represent the values obtained by pre-treatment with the compounds alone and black bars represent values obtained from cells pre-treated with the respective compound in combination with capsazepine. *CLDN1* was measured using RT-qPCR and IL-8 release was measured using ELISA. Data presented as fold change of the control pre-treatment without capsazepine. (Statistics: mean + SEM; technical replicates: 3, biological replicates: 5; **(a)** Mann–Whitney *t*-test **(b)** unpaired *t*-test or **(c–e)** Welch’s *t*-test, *p < 0.05, ***p < 0.001).

To test whether these achieved effects were TRPV1-dependent, compounds **4**, **14** and **15** were applied as a 24 h pre-treatment in a concentration of 10 µM and in combination with 1 µM of the TRPV1 antagonist capsazepine (Cervantes Recalde et al., 2024). Compounds **4** and **15** increased *CLDN1* gene expression the most among the 16 evaluated compounds, whereas compound **14** attenuated IL-8 expression and release. Co-treatment with the TRPV1 antagonist capsazepine did not downregulate the *CLDN1* expression caused by any of the compounds, but **14** and **15** showed an additive effect by a 1.08 ± 0.02 and 1.30 ± 0.07-fold increase compared to cells that were treated with the compounds solely (Figure 3a–c). IL-8 release was not altered by the addition of capsazepine to the pre-treatment (Figure 3d–f). As a result, the regulation of *CLDN1* gene expression by **4**, **14** and **15** is not dependent on the TRPV1 channel nor is the decrease in IL-8 release by **14**. Homovanillic acid esters, therefore, regulate *CLDN1* expression through an inflammatory pathway, possibly connected but not reliant on TRPV1 activation.

### 3.4 Regulation of *CLDN1* expression correlates with the expression of differentiation marker *IVL* but not with the basal keratinocyte marker *KRT14*

**FIGURE 4:**
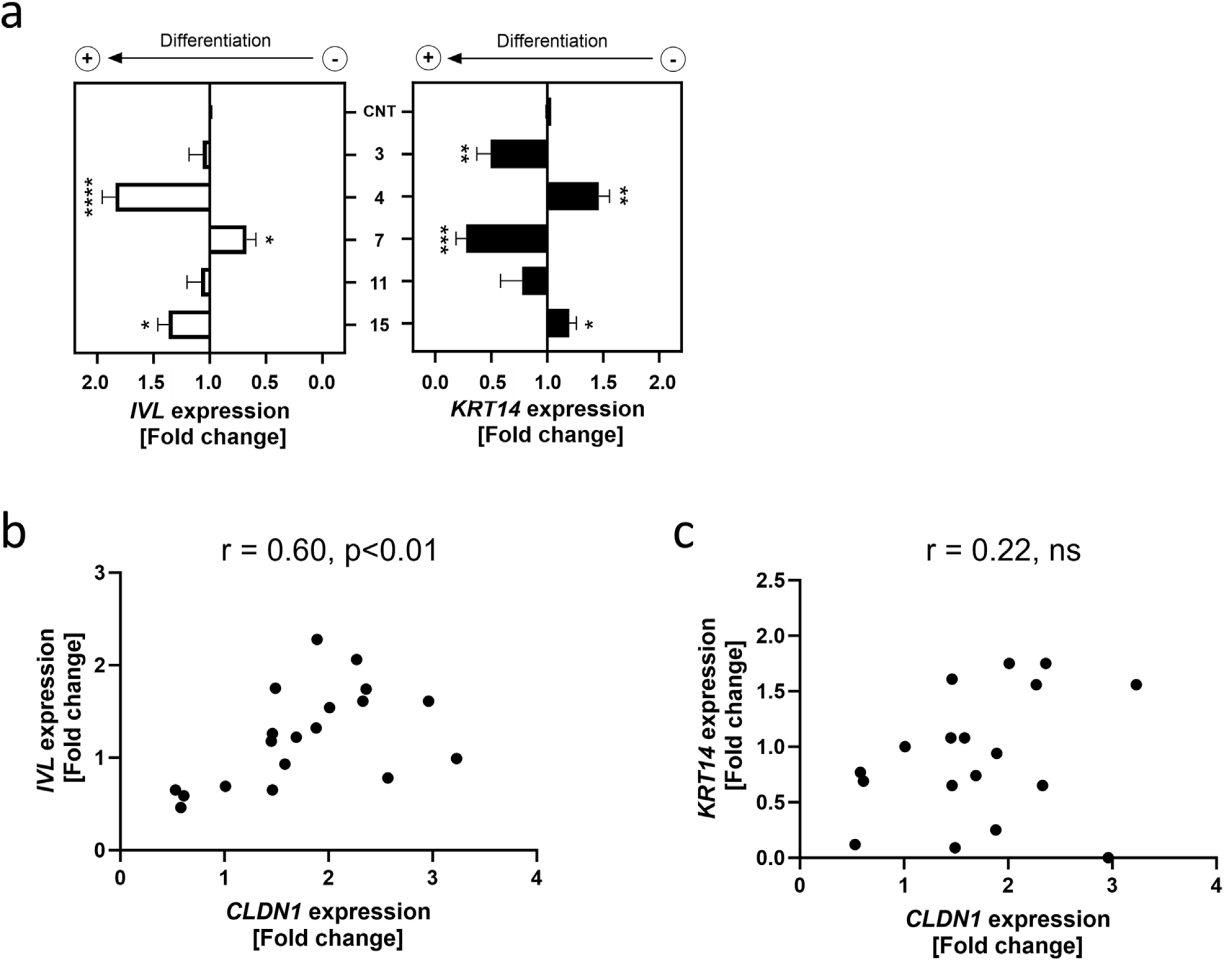
*CLDN1* expression correlates to differentiation marker gene expression. **(a)** *IVL* and *KRT14* gene expression after a 24 h treatment with compounds **3**, **4**, **7**, **11** and **15** at a common concentration of 10 µM or without pre-treatment (CNT) followed by a 6 h treatment with 20 ng/mL TNFα. Data presented as the fold change to the non-pretreated TNFα control (CNT). **(b)** Correlation plot of *IVL* expression against *CLDN1* expression **(c)** Correlation plot of *KRT14* expression against *CLDN1* expression. *IVL* and *KRT14* gene expressions were measured using RT-qPCR. [Statistics: mean + SEM; technical replicates: 3, biological replicates: 4; **(a)** Brown-Forsythe and Welch’s ANOVA with Dunnett’s T3 multiple comparisons *post hoc* test, *p < 0.05, **p < 0.01, ***p < 0.001, ****p < 0.0001; **(b,c)** Pearson correlation].

The NF-κB pathway is activated by TNFα during inflammation and is known to regulate keratinocyte differentiation (Banno et al., 2005). Because TJs are a characteristic of differentiated keratinocytes populating the SG (McGrath et al., 2010), we investigated whether the increase in *CLDN1* expression may be sign of the keratinocytes shifting towards a differentiated state. Two well-documented markers of differentiation used in HaCaT cells are *IVL* and *KRT14* (Watt, 1983; Fuchs, 1993; Ghahary et al., 2001). Whilst *IVL* expression increases in keratinocytes that have left the basal epidermal layer, *KRT14* is limited to basal layer keratinocytes (Watt, 1983; Fuchs, 1993). These markers were selected to assess the tendency towards differentiation of HaCaT keratinocytes after establishing the effect of a 6 h TNFα treatment (20 ng/mL) on their gene expression (Supplementary Figure S3). This was followed by the evaluation of a 24 h pre-treatment with four of the compounds that upregulated *CLDN1* expression (*i.e.* **3**, **4**, **11** and **15**) and one compound that downregulated *CLDN1* expression (i.e. **7**), respectively, and the results were calculated as fold change to the TNFα control (Figure 4a).

The SG differentiation marker *IVL* was upregulated 1.84 ± 0.12-fold by a pre-treatment with **4** and 1.37 ± 0.10-fold by a pre-treatment with **15** compared to the TNFα control. Compound **7** downregulated *IVL* expression to 0.67 ± 0.08-fold of the TNFα control. Contrastingly, the basal layer marker *KRT14* was significantly upregulated by a 1.46 ± 0.10-fold change to the TNFα control in cells pre-treated with **4** and downregulated in cells treated with **3** and **7** to a fold change of 0.50 ± 0.12 and 0.28 ± 0.09 of the TNFα control, respectively. There was a significant correlation between *CLDN1* and *IVL* gene expression with a r_s_ value of 0.60 (p < 0.01), however, no association between *CLDN1* expression and *KRT14* was found (Figures 4b,c). These results show that aliphatic tail modifications of homovanillic acid esters had an effect on *CLDN1* expression which is associated with changes in the differentiation marker *IVL,* but not *KRT14*.

## 4 Discussion

The TJ protein CLDN1 is essential for the formation of the TJ in the SG and for their role in epidermal permeability and therefore constitutes an important target for skin barrier recovery (Sugawara et al., 2013). During inflammation, *CLDN1* is upregulated presumably as a consequence of the promotion of differentiation elicited by the TNFα-mediated activation of the NF-κB pathway (Cervantes Recalde et al., 2024; Banno et al., 2005; Bhat et al., 2016) and can be further potentiated by activation of the TRPV1 channel (Cervantes Recalde et al., 2024). Although promising, capsaicin and other known TRPV1 ligands may trigger an undesired pain response through their action on neuronal TRPV1 channels present in the skin. The identification of structural components that support CLDN1 production without eliciting negative side effects can pave the way for the development of safe, non-irritating active compounds for the promotion of the skin barrier function. This study focused on the potential of homovanillic acid esters, non-pungent capsaicin analogues, to alter the expression of *CLDN1* in keratinocytes stimulated with TNFα. A total of 16 compounds were selected according to the “head, neck and tail” structural moieties characteristic of TRPV1 ligands (Caballero, 2022; Yang et al., 2015), and analyzed based on the modifications that potentially contribute to higher levels of *CLDN1* expression in HaCaT keratinocytes of basal epidermal layer morphology. Compounds **1**-**16** all shared an homovanillic acid ring as “head” and an ester bond as “neck” but the “tail” structures vary according to length, ramifications and double bonds. We hypothesized that structural features in the tail of the compounds are related to higher levels of *CLDN1* expression in undifferentiated HaCaT keratinocytes when used as a 24 h treatment preceding 6 h TNFα-mediated inflammation and this increase is independent of TRPV1.

In a previous study, we demonstrated that a 24 h pre-treatment with 10 µM capsaicin before TNFα-induced inflammation reduced the release of the inflammatory cytokine IL-8 after 6 h by approximately 40% and this was accompanied by a marked increase in *CLDN1* gene expression (Cervantes Recalde et al., 2024). To test whether these effects could be also achieved using less pungent structurally related compounds, we investigated the impact on *CLDN1* and *CXCL8* expression as well as IL-8 release of a 24 h pre-treatment with the homovanillic acid esters **1**-**16** with different aliphatic tail structures varying in lengths, ramifications and double bonds. Regulation of *CLDN1* was evaluated 6 h after induction of inflammation by TNFα. Baseline levels of TNFα had indeed strong increases in the expression of *CLDN1* and *CXCL8* already at 6 h after induction (Supplementary Figure S2). The shortest tail structure had 1 C-atom (**1**) whilst the largest structure had 8 C-atoms in the carbon chain (**14**). Out of the 16 homovanillic acid esters, 10 modulated *CLDN1* expression. Compounds **2, 5, 7** and **8** reduced *CLDN1* expression, whereas compounds **1**, **3**, **4**, **11**, **12**, and **15** upregulated its expression. *CXCL8* expression was upregulated by compounds **1**, **3**, **11, 12** and downregulated by compounds **7** and **14**. Compounds **7** and **14** also reduced IL-8 release. When SAR analysis was conducted to identify structural components in the hydrophobic tail of the homovanillic acid esters, it was discovered that the most conducive structural characteristic towards higher levels of *CLDN1* expression was a tail size of 5 or 6 C-atoms with 6 or 7 rotatable bonds. Modifications in the tail that reduced the size or amount of rotatable bonds to these numbers potentiated *CLDN1* expression. Similar effects were found with the addition of ramifications or double bonds in the third position after the ester bond. *CXCL8* was predominantly upregulated by compounds with 4 C-atoms in the main aliphatic chain or 5 total C-atoms in the tail structure. The largest compound, **14**, was also the compound that downregulated *CXCL8* and IL-8 release the most out of all compounds. Therefore, homovanillic acid ester structures targeting *CLDN1* upregulation might benefit from a design with a shorter aliphatic tail, whereas structures targeting *CXCL8* downregulation would require larger tail moieties to avoid enhancing the expression of this gene during TNFα induced inflammation.

However, short aliphatic tails in TRPV1 ligands result in a reduced affinity to the channel (Gavva et al., 2004), raising the possibility that the TRPV1 channel is not involved in eliciting the observed effects. To evaluate this, the participation of the channel was tested by using the TRPV1 antagonist capsazepine in combination with compounds **4** and **15**, which upregulated *CLDN1* expression, and compound **14** which reduced IL-8 release after induction of inflammation with TNFα. The participation of TRPV1 in these effects could not be verified as the addition of capsazepine to the compound pre-treatment did not counteract the effect elicited by the compound alone but potentiated the increase in *CLDN1* expression even further. The additive effect on *CLDN1* expression seen in cells due to capsazepine suggests that the TRPV1 antagonist may contribute to the regulation of *CLDN1* through a synergistic mechanism extending beyond direct TRPV1 channel interaction. Further experiments using a TRPV1 knock out or a gene silencing model can deliver more accurate information on the participation of the channel in these effects.

**FIGURE 5:**
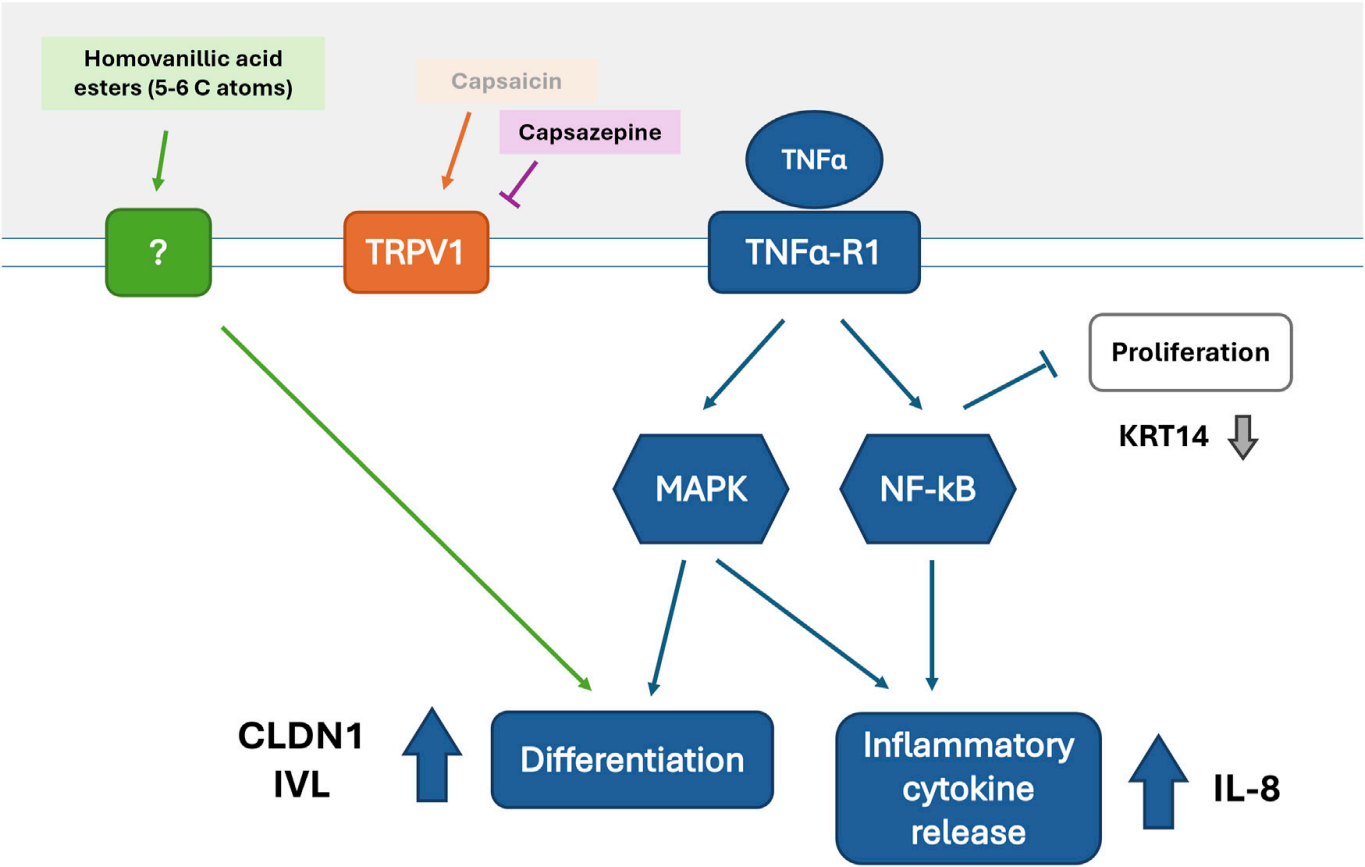
Potential mechanism of action for homovanillic acid esters leading to increased CLDN1 expression during TNFα induced inflammation.

By further considering the gene expression tendencies shown by the selected compounds it was discovered that *CXCL8* and *CLDN1* expression showed a moderate correlation. This correlation could be explained by their downstream position to TNFα in the NF-κB and mitogen-activated protein kinase (MAPK) pathways (Figure 5) (Chen et al., 2018; Sabio and Davis, 2014; Webster and Vucic, 2020). TNFα activates the NF-kB pathway in keratinocytes, mediating the release of pro-inflammatory cytokines such as IL-8 (Chen et al., 2018; Banno et al., 2005). The MAPK pathway is also activated leading to pro-inflammatory cytokine expression and cell differentiation accompanied by TJ protein expression (Qi and Elion, 2005; Bongki and Breton, 2016). The alterations in *CLDN1* expression induced by homovanillic acid esters during inflammation, therefore, could be attributed to enhanced differentiation of keratinocytes due to the necessary production of TJ proteins like CLDN1 in the burgeoning SG. To investigate this, we selected markers for differentiation (*IVL)* and proliferation (*KRT14*) and assessed whether the homovanillic acid esters promoted differentiation or whether they reinforced the proliferative state. IVL is a precursor protein of the cornified envelope (or SC) and is found predominantly in the outer layer of skin biopsies (Watt, 1983; Murphy et al., 1984). KRT14 is a widely used marker to identify cells in basal layers of the skin that are not undergoing differentiation but are rather more likely to remain in the basal layer and undergo proliferation (Fuchs, 1993). The baseline values of a TNFα treatment for 6 h correspond to an increased *IVL* expression compared to the untreated control as well as a downregulation of *KRT14* expression vs. untreated control consistent with the previously documented effect of TNFα in keratinocytes where differentiation is promoted and proliferation inhibited (Banno et al., 2005) (Supplementary Figure S3). After pre-treatment with selected homovanillic acid compounds, *IVL* was downregulated by **7** as well as upregulated by **4** and **15**, corresponding to the tendency of these compounds to alter *CLDN1* expression in comparison to the TNFα treatment as control. On the other hand, *KRT14* expression was upregulated by compound **4** and downregulated by compounds **3** and **7.** The expression of *IVL* was correlated to the expression pattern of *CLDN1*, but *KRT14* did not show any correlation. This suggests that the regulation of *CLDN1* expression by compounds **7**, **4** and **15** acts on components of the NF-κB and MAPK pathways related to keratinocyte differentiation (Banno et al., 2005; Bongki and Breton, 2016; Li et al., 2021; Shin et al., 2013).

Considering all this, we propose a possible mechanism of action (Figure 5) where upregulation of *CLDN1* and *CXCL8* is seen during inflammation induced by TNFα through the activation of the NF-κB and MAPK pathways and the consequential increase in inflammatory cytokine release (e.g., IL-8) and differentiation (Sabio and Davis, 2014; Webster and Vucic, 2020). The pathways leading to differentiation are strengthened by the inhibition of keratinocyte proliferation through NF-κB’s action (Takao et al., 2003). The increase in differentiation promotes TJ protein expression (CLDN1) as well as the expression of other proteins required to form the cornified envelope or SC (e.g., IVL). On the other hand, downregulation of proliferative activity leads to decrease or stagnation of the basal production of keratins (e.g., keratin 14) by keratinocytes. Treatment with homovanillic acid esters with aliphatic tails of 5–6 C-atoms intervenes components regulating differentiation downstream of TNFα by an alternative mechanism that does not require TRPV1 activation resulting in increased expression of *CLDN1*. Future studies should explore this hypothesized mechanism by investigating individual components of the NF-kB and MAPK pathways.

The evaluation of the structural features of the homovanillic acid esters in this study led to non-pungent capsaicin analog structures that promoted *CLDN1* expression and provided information on characteristic features that lead to elevated gene expression levels of this TJ protein. Our findings on *CLDN1* promotion by these compounds show that comparable effect sizes to those of capsaicin can be achieved (Cervantes Recalde et al., 2024), but direct comparisons of capsaicin and homovanillic acid esters would be needed to confirm the effectivity of the compounds in relation to well-known TRPV1 ligands. This, in combination with a broader selection of structural modifications in the homovanillic acid ester molecule as well as metabolites stemming from esterase activity would enable a more holistic picture on which molecular characteristics lead to targeted modulation of CLDN1. This study focused exclusively on the effects of the selected homovanillic acid esters on keratinocytes after a 6-h pre-treatment with TNFα, leaving the effects of the individual compounds on untreated keratinocytes unexplored. However, the increases of *CXCL8* and *CLDN1* expression due to the TNFα treatment were overwhelmingly superior to that of capsaicin (Supplementary Figure S2). This suggests that the effect size of homovanillic acids on healthy keratinocytes might be overpowered by TNFα, but this needs to be confirmed through experimentation.

Furthermore, the study design aimed to study compounds with modifications that purposedly reduce the affinity for the TRPV1 channel in order to determine whether non-pungent compounds affected *CLDN1* gene expression during inflammation. This marker was used for its robustness and prevalence in both inflammation and TJ function (Furuse et al., 2002; Bergmann et al., 2020; Arnold et al., 2024). Nevertheless, we recognize the need for further research on a wider variety of TJ proteins, in particular ZO-1 and claudin-4, to further substantiate these findings and provide a more comprehensive understanding of the effect of homovanillic acid ester pre-treatment on TJs. This is also required regarding the use of *IVL* and *KRT14* as markers for keratinocyte differentiation versus proliferation, which could be complemented by the inclusion of other differentiation markers like transglutaminase-1, loricrin or filaggrin. It is also important to mention that a 6 h timeframe is likely not long enough to verify keratinocyte differentiation with morphological changes and protein abundance measurements. In our previous work, CLDN1 protein abundance changes were only verifiable 48 h after induction of TNFα, and morphological changes regarding differentiation were limited (Cervantes Recalde et al., 2024; Saha et al., 2022). Longer incubation times would be required in future to explore the mechanisms that lead to the effects of homovanillic acid ester treatment on keratinocyte differentiation during inflammation. Notwithstanding this, we do believe a closer look towards the effects of phenolic compounds on keratinocyte differentiation needs to be taken, because of the promising data presented in this and other studies (Petersen et al., 2011; Wang et al., 2014).

This study provides novel evidence for homovanillic acid esters with characteristic structural motifs, e.g., 5 or 6 C-atoms in the aliphatic ester tail, to have the potential to support skin barrier recovery and TJ development. The suggested intervention of homovanillic acid esters in differentiation-related pathways invites an extended investigation and further characterization *in vitro* and *ex vivo* experiments could lead the discovery of important molecules for effective skin repair treatments that can bypass capsaicin’s adverse effects.

In conclusion, the structure-activity relationship between homovanillic acids and *CLDN1* expression was determined in HaCaT keratinocytes of basal layer morphology at early stages of TNFα-induced inflammation. *CLDN1* expression was modulated by several of the tested homovanillic acid esters without involvement of the TRPV1 channel. The structural characteristics of compounds that upregulated *CLDN1* expression were 5 or 6 C-atoms in the aliphatic tail of the ester with ramifications or double bonds in the third C-atom after the ester bond. Because of the short length of the aliphatic tail, it is possible that the affinity for the TRPV1 channel is reduced in comparison with known TRPV1 ligands. Nevertheless, an alternative NF-κB pathway regulation by the compounds targeting differentiation of HaCaT keratinocytes as evidenced by the increase in *IVL* expression is conceivable. This gene is not expressed in basal keratinocytes but was induced in correlation to the expression of *CLDN1* advocating for differentiation towards SG morphology where TJs are formed. This presents a first hint on the potential of homovanillic acid esters to aid in epidermal barrier recovery.

## Data Availability

The original contributions presented in the study are included in the article/Supplementary Material, further inquiries can be directed to the corresponding author.
