# Supplementary material for: Role of homovanillic acid esters in the regulation of skin inflammatory pathways and their effect on tight junction protein expression
Source: Front Pharmacol. 2025 Jul 21;16:1629941. doi: 10.3389/fphar.2025.1629941 (PMC12319341; doi:10.3389/fphar.2025.1629941)
Supplement: Supplementary file 1 [file DataSheet1.docx]

Supplementary Material

Role of homovanillic acid esters in the regulation of skin inflammatory pathways and their effect on tight junction protein expression

Maria Fernanda Cervantes Recalde^1,2^, Elena Zoe Bogensperger^1^, Joachim Hans^3^, Dominik Stuhlmann^3^, Veronika Somoza^1,4^, Barbara Lieder^1,5*^

^1^Institute of Physiological Chemistry, Faculty of Chemistry, University of Vienna, Vienna, Austria

^2^Vienna Doctoral School in Chemistry (DoSChem), University of Vienna, Vienna, Austria

^3^Symrise AG, Muehlenfeldstrasse 1, Holzminden, Germany

^4^Leibniz Institute of Food Systems Biology, Technical University of Munich, Freising, Germany

^5^University of Hohenheim, Institute of Clinical Nutrition, Stuttgart, Germany

*** Correspondence:**Corresponding Author
Barbara.Lieder@uni-hohenheim.de

Keywords: skin, inflammation, TRPV1_3_, claudin 1, homovanillic acid ester

**1 Supplementary Figures**


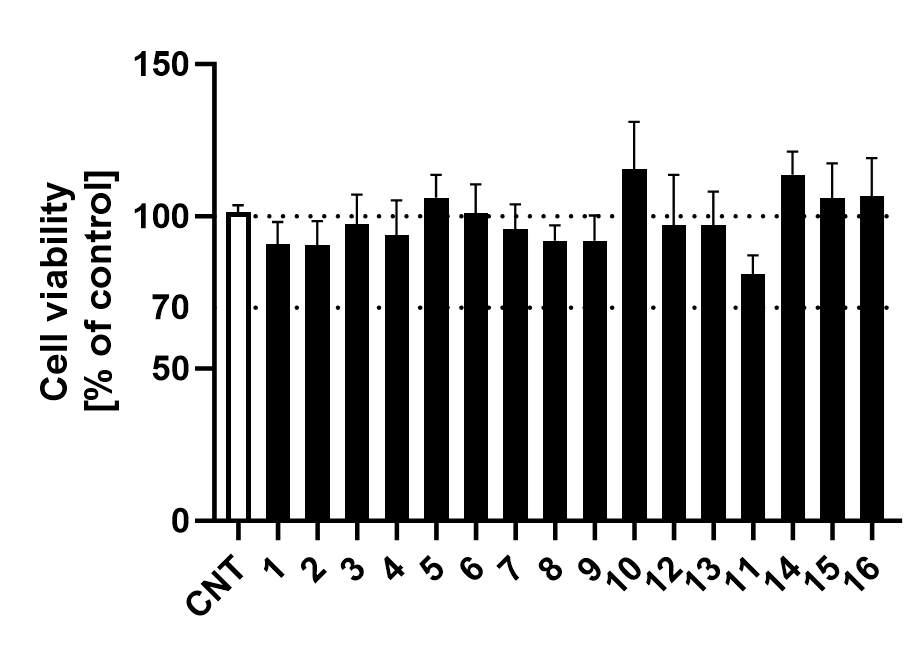


FIGURE S1. Cell viability assessment of HaCaT cells pre-treated with 10 µM of the different homovanillic acid esters for 24 h. No significant decrease in cell viability was observed after 24 h application of compounds **1**-**16** (black). Data presented as percentage of the non-treated control (white). Dotted lines represent 100 % cell viability and the 70 % cut-off for assessing adverse effects on cell viability measured using an MTT assay. (Statistics: mean + SEM; technical replicates: 4, biological replicates: 5, ordinary one-way ANOVA)


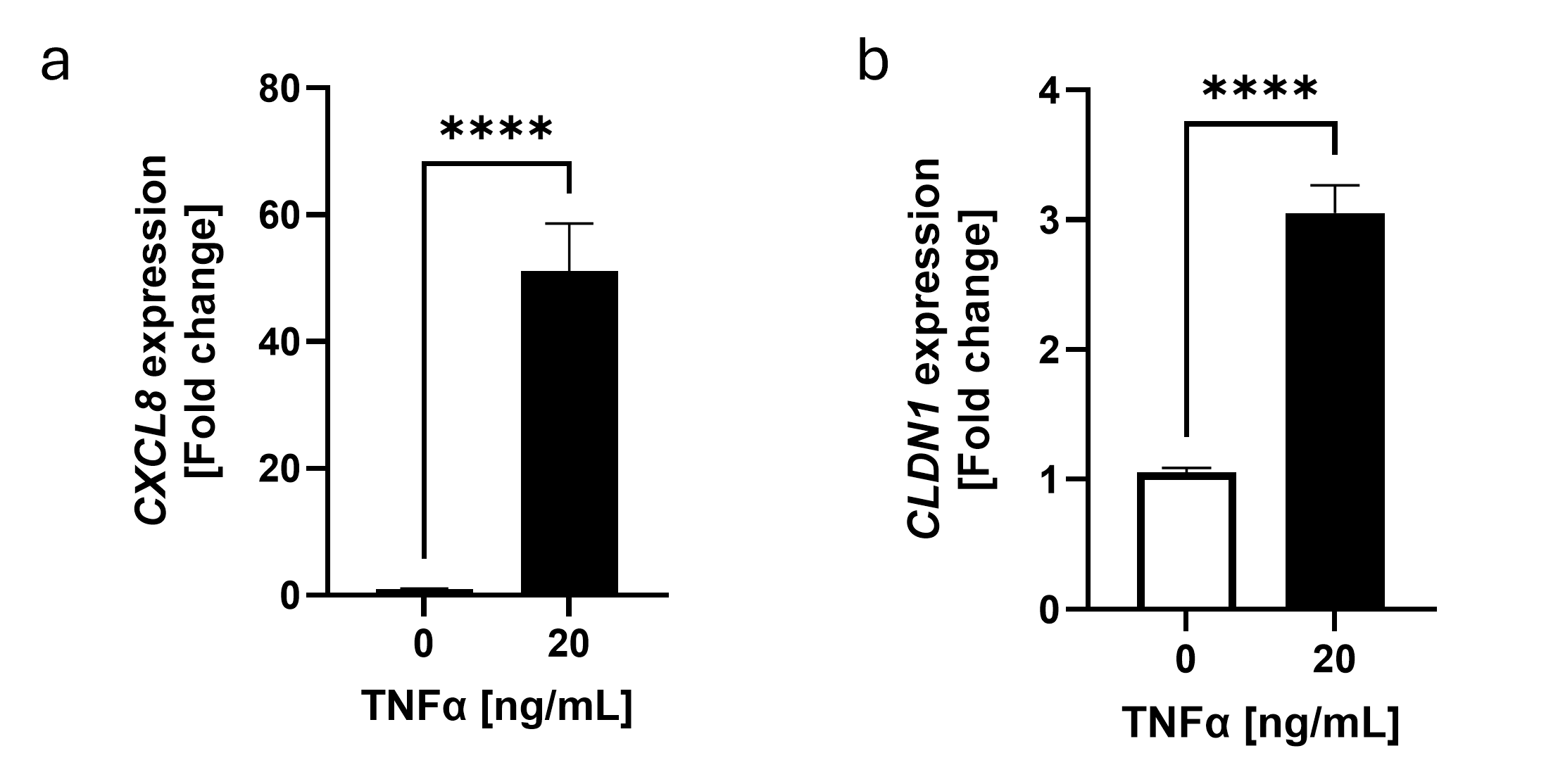


FIGURE S2. Characterization of *CXCL8* and *CLDN1* gene expression in skin samples after TNFα treatment for 6 h. HaCaT keratinocytes cultured under low calcium conditions (0.06 mM Ca^2+^) for 4 days were treated with 20 ng/mL TNFα for 6 h. (a) *CXCL8* and (b) *CLDN1* gene expression (black) was measured after TNFα treatment and presented as fold change of the non-treated control (white). Relative gene expression was measured with RT-qPCR and normalized to the geomean of the reference genes (Statistics: mean + SEM; technical replicates: 3, biological replicates: 5).


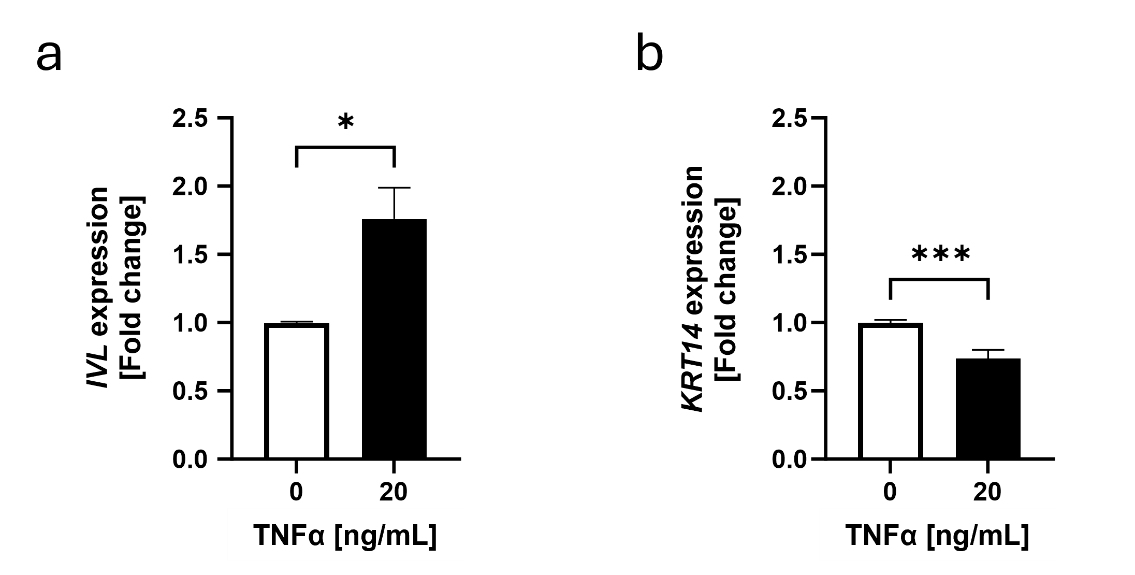


FIGURE S3. TNFα-induced changes in *IVL* and *KRT14* expression in HaCaT keratinocytes. TNFα increased (a) *IVL* gene expression and (b) reduced *KRT14* after 6 h treatment with 20 ng/mL TNFα compared to the non-treated control. *IVL* and *KRT14* expression were measured using real-time PCR. Data is presented as fold change to non-treated control. White bars represent the non-treated control and black bars represent TNFα treatment. (Statistics: mean + SEM; technical replicates: 3, biological replicates: 10-11; Welch’s t- test, *p<0.05, ***p<0.001).


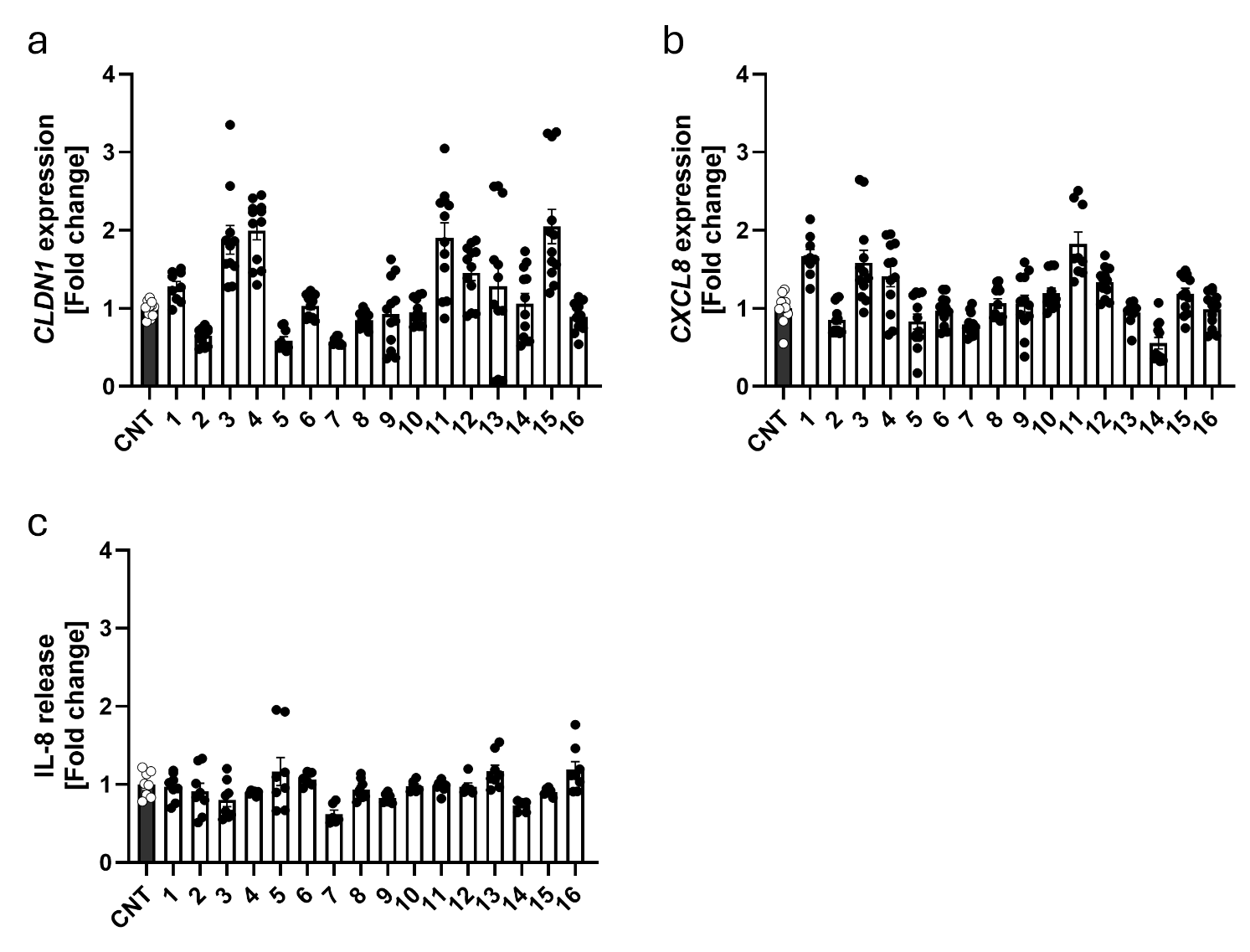


FIGURE S4. TNFα-induced variation of *CLDN1* and *CXCL8* expression in HaCaT keratinocytes. (a) *CLDN1* expression, (b) *CXCL8* expression and (c) IL-8 release corresponding to the data presented in figures 2a and 2b. Mean value + SEM (bars) and single datapoints (dots) are presented for the TNFα control and the homovanillic acid esters (**1**-**16**). Data is presented as fold change to TNFα control. (Statistics: mean + SEM; technical replicates: 3, biological replicates: 4).


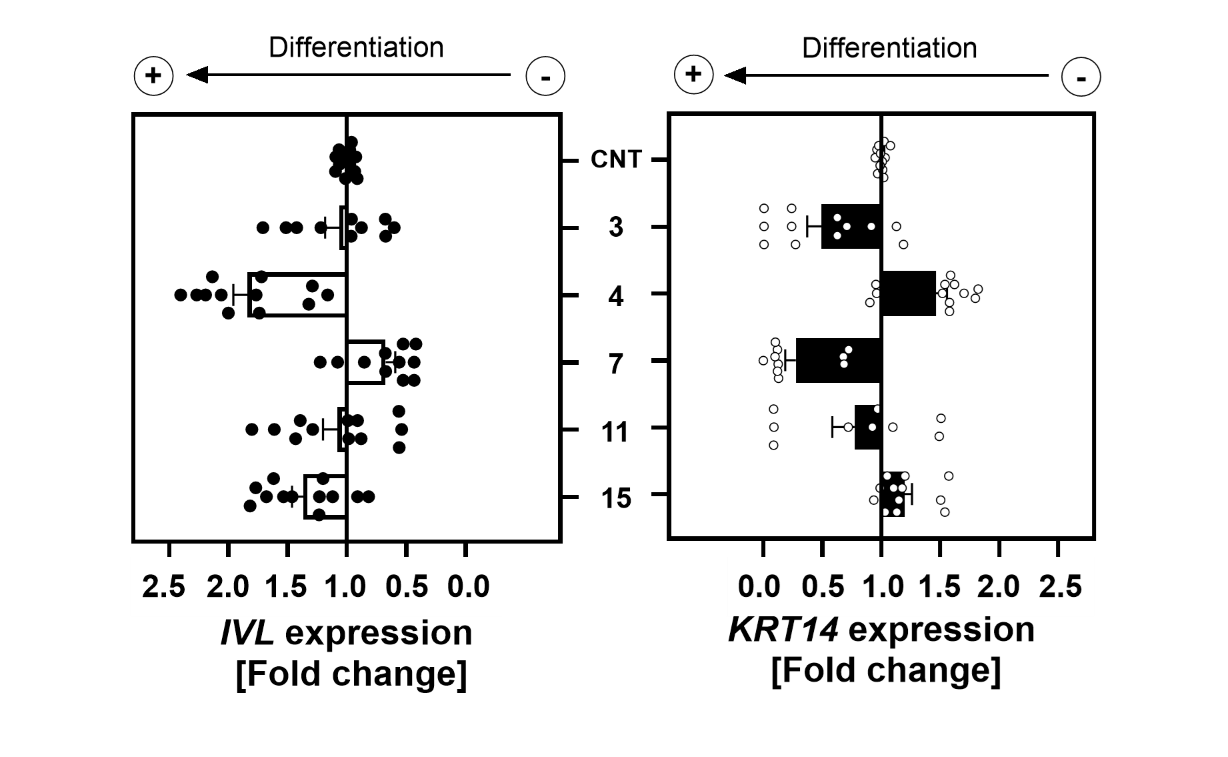


FIGURE S5. TNFα-induced variation in *IVL* and *KRT14* expression in HaCaT keratinocytes corresponding to the data presented in figures 4a. Mean value + SEM (bars) and single datapoints (dots) are presented for the TNFα control and compounds **3**, **4**, **7**, **11**, and **15**. Data is presented as fold change to TNFα control. (Statistics: mean + SEM; technical replicates: 3, biological replicates: 4).
